# Reversible optical tuning of GeSbTe phase-change metasurface spectral filters for mid-wave infrared imaging

**DOI:** 10.1364/OPTICA.392878

**Published:** 2020-07-01

**Authors:** Matthew N. Julian, Calum Williams, Stephen Borg, Scott Bartram, Hyun Jung Kim

**Affiliations:** 1 Charles L. Brown Department of Electrical and Computer Engineering, University of Virginia, Charlottesville, Virginia 22904, USA; 2 National Institute of Aerospace, Hampton, Virginia 23666, USA; 3 Department of Physics, Cavendish Laboratory, University of Cambridge, Cambridge, CB3 0HE, UK; 4 NASA Langley Research Center, Hampton, Virginia 23666, USA

## Abstract

Tunable narrowband spectral filtering across arbitrary optical wavebands is highly desirable in a plethora of applications, from chemical sensing and hyperspectral imaging to infrared astronomy. Yet, the ability to reconfigure the optical properties, with full reversibility, of a solid-state large-area narrowband filter remains elusive. Existing solutions require either moving parts, have slow response times, or provide limited spectral coverage. Here, we demonstrate a 1-inch diameter continuously tunable, fully reversible, all-solid-state, narrowband phase-change metasurface filter based on a GeSbTe-225 (GST)-embedded plasmonic nanohole array. The passband of the presented device is ∼
74nm with ∼
70%
 transmittance and operates across the 3–5 µm thermal imaging waveband. Continuous, reconfigurable tuning is achieved by exploiting intermediate GST phases via optical switching with a single nanosecond laser pulse, and material stability is verified through multiple switching cycles. We further demonstrate multispectral thermal imaging in the mid-wave infrared using our active phase-change metasurfaces. Our results pave the way for highly functional, reduced power, compact hyperspectral imaging systems and customizable optical filters for real-world system integration.

## 1. INTRODUCTION

Narrowband spectral filtering is integral in applications ranging from chemical spectroscopy and hyperspectral imaging to infrared (IR) astronomy [1,2]. For all major applications, multilayer interference (dichroic) filters offer unrivaled optical performance characteristics, yet unfortunately are passive components. For tunable optical properties—critical for probing more than a single wavelength and wide waveband operation—tunability is effectively mimicked using motorized filter wheels containing several narrowband filters, or through mechanical tuning mechanisms [3]. Alternative frameworks for active tuning include Fabry–Perot-based micro-electro-mechanical systems (MEMS) [4–7], liquid crystal (LC) tunable filters [8,9], and acousto-optical tunable filters [10]. Nevertheless, all approaches suffer from varying inherent limitations, such as having moving parts; being complex/expensive to manufacture; offering slow response times; and providing limited spectral bandwidth/resolution. For compact, fast-switching, narrowband spectral filtering across wavebands, no single solution currently exists.

In recent years, nanophotonic-inspired approaches, such as plasmonic nanostructure arrays and metasurfaces, have been proposed as potential solutions for tunable/reconfigurable spectral filtering [11–17]. Through design, their optical response can be tailored to specific wavebands and thus specific applications. To date, these devices have been primarily passive, i.e., their spectral response fixed post-manufacture. In contrast, tunable nanophotonic approaches, such as active metasurfaces, are capable of on-the-fly dynamically tunable operation and have recently been demonstrated to show reconfigurable spectral filtering, thermal emission, beam steering, and tunable metalenses [11,13,15,16,18–23]. A number of different approaches have been utilized, including thermally/electrically tunable structures based on ${VO}_{2}$ phase changes [5,21]; multi-quantum well (MQW) structures [24–27]; LCs [9,28–30]; MEMS [4,6,7,31]; and epsilon-near-zero (ENZ) materials [32–34]. Nevertheless, translating these designs into multiple spectral bands is challenging. ENZ structures, for example, require operation at their respective ENZ wavelengths, which inherently limits their operational spectrum. ${VO}_{2}$ has seen widespread utility as a phase-change material (PCM) in various spectral wavebands [5,21] but is not sufficiently transparent in the metallic state to be used for highly transmissive, real-world applications such as imaging or remote sensing, and is primarily suited for applications involving the modulation of a device’s reflective or absorptive properties. LCs suffer from a similar drawback; their organic nature results in strong vibrational absorption bands in the mid-wave IR (MWIR), limiting their utility across multiple wavebands. Additionally, LC-based nanophotonic color filters are generally limited in their tunability, bandwidth, and efficiency, and are thus often utilized in reflective color generation [9,28–30]. MQW structures have been demonstrated in an array of tunable photonic applications [24–27]. However, MQWs rely on metallic contacts to tune their effective refractive index (optical response), and hence are more suited to reflective/absorptive applications, and are also not generally suited for the visible or near-IR spectrum. Tunable transmission filters using graphene plasmonic ribbons have been demonstrated recently [35]. However, a wide bandwidth, low transmission efficiency (∼
10%
), and reduced long-term chemical stability make them equally unsuitable for the majority of real-world applications. Additionally, the reliance on the plasmonic resonance of graphene restricts these designs to IR/THz operation.

PCMs based on transition metal chalcogenide alloys such as GeSbTe (GST) are largely transparent across various spectral imaging wavebands and exhibit significantly large, reversible refractive index modulation upon crystallization, making them ideal candidates for use in such spectrally robust filter designs [36]. Such “exotic” optical materials have been utilized recently as the tunable medium for absorptive color filters [37–40], thermal emission switches [18,19], MWIR absorptive pixel arrays [41], programmable metasurface phase and amplitude modulators [42,43], and tunable metalenses [22,23]. GST has a dielectric permittivity of ∼
9.0 and ∼
25.0 in its amorphous and crystalline states, respectively. Hence, GST provides an attractive tuning mechanism for resonance-based devices, whereby the surrounding index strongly controls the spectral position of the resonance. Moreover, crystalline GST (c-GST) exhibits a non-negligible increase in its extinction coefficient compared to amorphous GST (a-GST), which has thus far limited its use in transmissive applications. Other comparable PCMs, such as GeSbSeTe (GSST), have recently been proposed as low-loss alternatives to GST but are fundamentally limited by slow (>
ms) switching times compared to the ns/ps response of GST [44].

GST switching operation is generally considered binary in nature; operating in either its a-GST or c-GST state, subsequently limiting prospective devices to dual-modal operation [45] as opposed to fully continuous. Theoretical studies have proposed absorptive optical devices based on partial crystallizations of GST [37]. Other preliminary experimental studies have shown optical devices with some level of control using partial crystallinities; however, thus far, no reversible switching operation has been realized with sub-optimal filtering properties (i.e., broad passbands) and limited utilization/integration of GST (i.e., GST deposited only within small volumes of the optical design) [46].

In this work, we present for the first time, phase-change tunable MWIR metasurface spectral filters based on GST-embedded plasmonic nanohole arrays (PNAs) with high transmission efficiency, narrowband performance, a continuous tuning range, and fully reversible operation. Reconfigurability is achieved through accessing a continuum of partial-GST crystallinities using single nanosecond laser-pulse-induced phase transformation. These partial crystallinities—previously unexploited/under-utilized experimentally—exhibit repeatable, stable behavior across a number of phase change cycles. Our results show excellent optical performance characteristics: high transmittance (∼
70%
) on resonance, narrow bandwidth (∼
74nm) within the 3–5 µm (MWIR) waveband, and near perfect reflection off-resonance. The devices are able to both operate in transmission mode and offer narrowband performance due to the large field enhancement arising from the high-index GST being deposited inside the nanoholes, as opposed to a continuous, unpatterned film below the hole array. This high field enhancement appears to negate an increased extinction coefficient associated with c-GST, and the device geometry—with total PCM filling volume of the nanoholes—provides the necessary design novelty to enable best-in-class optical performance. We show real-world applicability through multispectral thermal imaging demonstrations by integrating our metasurfaces with a MWIR camera. Our device design framework can be tailored to operate across any optical waveband through phase-change alloy selection. The results presented represent a significant step toward robust, tunable spectral filters, with applications in compact, fast-switching, and all-solid-state tunable filter systems for imaging in a wide variety of fields, from astronomy to remote Earth sensing.

## 2. RESULTS

### A. GST Thin-Film Morphology

**Fig. 1.**
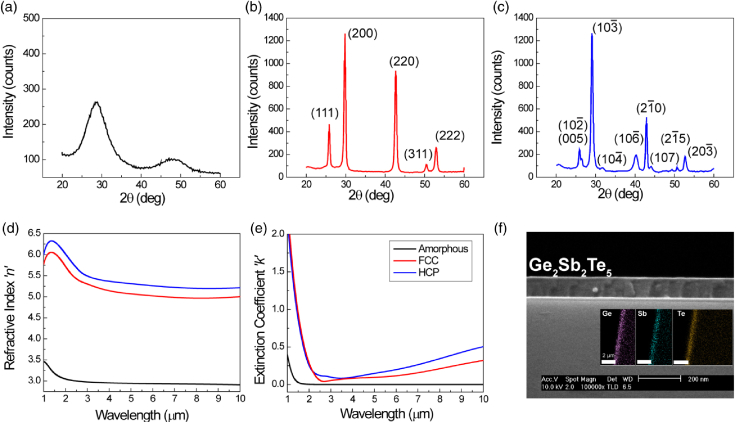
Thin-film GST characterization. XRD (a)–(c) and ellipsometry (d), (e) data for a-GST (black curves), FCC c-GST (red curves), and HCP c-GST (blue curves). The average Δ
n is ∼
2.0 between a-GST and c-GST, with HCP c-GST exhibiting slightly higher refractive index and extinction coefficient compared to FCC c-GST. (f) SEM cross-section image of the GST film deposited on $CaF_{2}$. The inset shows a top-view energy-dispersive x-ray spectroscopy (EDS) mapping of the film edge, showing the clear presence of Ge, Sb, and Te species.

Initially, to verify thin-film quality, $Ge_{2}Sb_{2}Te_{5}$ (GST-225) films were deposited onto $CaF_{2}$ substrates via RF magnetron sputtering from a GST-225 target. For low-loss phase-change electro-optical devices, PCM compounds with a large Δ
n and small Δ
k, in the waveband of interest, are desired. The choice of PCM stoichiometry requires careful consideration of the intended application area’s key operating requirements. In comparison to other popular GST stoichiometries, such as GST-326, GST-629, and GST-8211 [44,47], GST-225 was selected for the MWIR spectral filters in this work due to its ubiquity in commercial rewritable optical storage media; low power switching; fast-tuning speed; relative ease of conformal deposition; large Δ
n/Δ
k across the MWIR; and optimized process parameters established in our laboratory. Detailed descriptions of the GST deposition and characterization are given in Supplement 1, Section 1. The stoichiometric ratios of deposited films were confirmed via direct current plasma atomic emission spectrometry (DCP-AES) measurements. The crystallinity and film thicknesses were determined via x-ray diffraction (XRD) measurements and SEM of cleaved samples, respectively. The complex refractive indices of the films were characterized using IR-variable angle spectroscopic ellipsometry (J.A. Woollam, Co.) in the spectral range from 1–10 µm. Results of both the XRD and ellipsometry measurements of as-deposited GST-225 are shown in Fig. 1 for a-GST and both hexagonal-centered cubic (HCC) and face-centered cubic (FCC) c-GST. The XRD data [Figs. 1(a)–1(c)] show a clear transition from amorphous [Fig. 1(a)] to FCC and HCP states [Figs. 1(b) and 1(c)]. The two crystal states share the same main peak at $2\theta =30^{\circ }$, but show a stark difference in the magnitudes of the peaks centered around 42° and 26°, indicating the different GST unit cell configurations. The transition from a-GST to c-GST shows the expected large refractive index shift Δ
n=2.0, with a small increase in the extinction coefficient as wavelength increases [Figs. 1(d) and 1(e)] [48]. As can be seen in the characterization results in Fig. 1(f), the as-deposited films show very low surface roughness and high uniformity in thickness.

### B. Phase-Change Plasmonic Metasurface

**Fig. 2.**
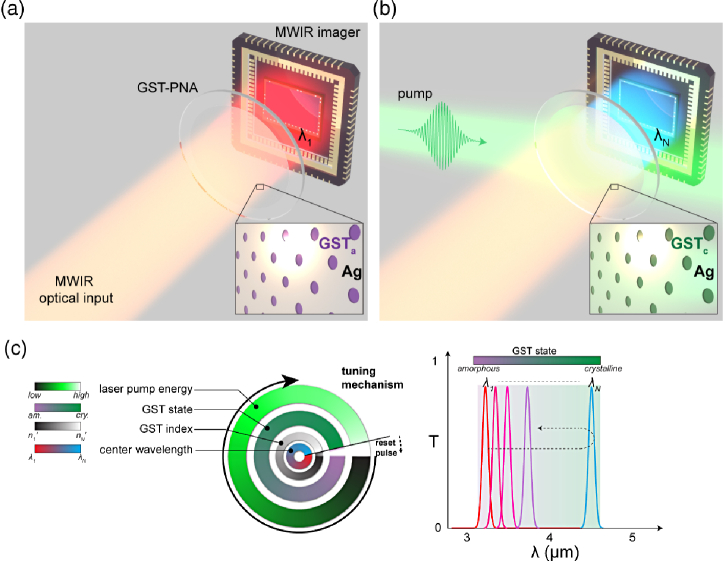
Device concept. Tunable GST-plasmonic nanohole array metasurface for the MWIR waveband. The MWIR optical input is imaged through GST-PNA filer in its initial, amorphous state (a), with initial center wavelength, λ
1. Through a laser pulse incident on the GST-PNA active area, the GST crystallinity is modified (phase change), and the resultant transmission response (center wavelength, with initial center wavelength, λ
N) is spectrally shifted (b). This behavior is summarized in (c), whereby the pump energy controls GST-state, which in turn changes its refractive index, hence spectrally shifts the center wavelength from the resonant PNA. A “reset pulse” returns the GST to its initial state, thus device to initial transmission center wavelength.

**Fig. 3.**
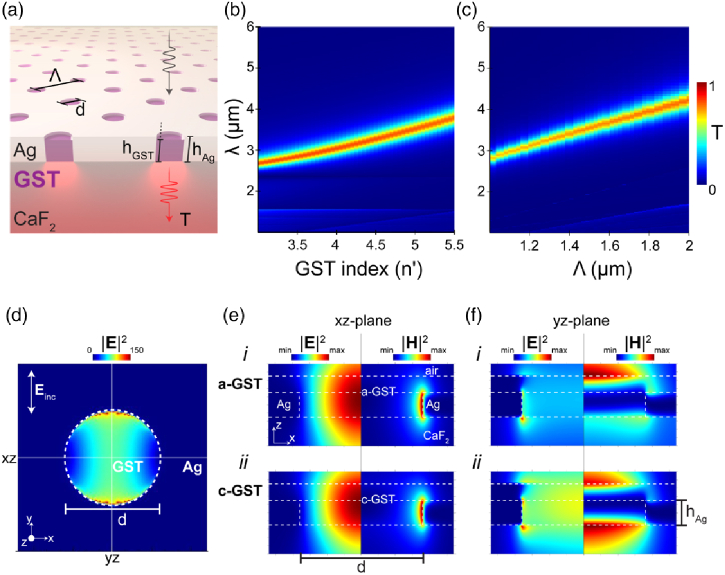
FDTD simulations of the optical response of the GST-PNA concept (a). Simulated transmission response of the GST-PNA device as a function of GST refractive index (b), and as a function of hole period (c), for $h_{Ag}=60\;nm$ metal film thickness and d=0.4×
Λ
. E-field plot (d) showing SPR-generated field enhancement on-resonance at the boundary between Ag/GST inside the PNA cavity. (e), (f) E- and H-field enhancement plots, on-resonance in respective states, of the GST-PNA at xz and zy cross-section slices of a single hole in an array, in amorphous (i) and crystalline (ii) GST states, where Λ
=period, d=hole diameter=720nm, and the experimentally derived GST complex refractive index data in Figs. 1(d) and 1(e) utilized inside the hole array. Source injection from $CaF_{2}$ side.

Our GST-based PNA MWIR metasurface filter concept is shown in Fig. 2. The extraordinary optical transmission (EOT) response, thus local field enhancement, of PNAs strongly depends on the surrounding index [49–52]. Typically, this is exploited by modulating the index above or below the hole array [9,28–30,35]. However, the resonance appears to be sensitive to the index inside the individual holes—a fact that has been unexplored experimentally [52]. Here, the GST-PNA metasurface response is therefore dependent on the crystallization state of GST, which is subsequently tuned (continuously) through differing partial crystallizations, using a ns laser pulse (Fig. 2). This operating principle is summarized in Fig. 2(c), whereby an increasingly large pump energy incident upon the active area continuously adjusts the GST crystallinity, changing its index, and hence spectrally shifting the surface plasmon resonance (SPR) mode and changing the resulting transmission (EOT) response of the device. A “reset pulse” (high energy) is then used to revert the device back to a-GST, thus allowing for repeatable operation. Using the experimentally measured complex refractive index of GST [Figs. 1(d) and 1(e)], electromagnetic simulations (Lumerical FDTD [53]) of the GST-PNA device, shown schematically in Fig. 3(a), were performed in order to optimize geometric parameters for narrowband optical performance across 3–5 µm. Ag is chosen due to its superior optical properties across the MWIR in comparison to other noble metals (e.g., Al, Au). A hexagonal array was chosen in order to decrease polarization sensitivity and increase overall space utilization. Additionally, hexagonal arrays have been shown to exhibit higher Q resonances compared to square arrays [54].

The hole diameter of the hexagonal array was optimized via sweeping the ratio of hole diameter (d) to period (Λ
) at a given period; a ratio of 0.4×
Λ
 was found to be optimal with Λ
=1800nm and 60 nm thickness ( Supplement 1, Fig. S5). The effect of array periodicity and embedded refractive index GST/Ag PNAs (with d=720nm) is presented in Figs. 3(b) and 3(c). As expected, a near linear dependence on both quantities is observed, and the simulated FWHM is consistently ∼
80nm throughout the simulated range (with a slight increase at longer resonance wavelengths), with low reflection shown off-resonance ( Supplement 1, Fig. S5), corresponding to a high out-of-band blocking region of optical density (OD) ∼
3−
4, competitive against established tunable filter technologies. The simulations performed show a tuning range of 2.9–4.1 µm; however, by further increasing/decreasing the period of the nanohole array, this range can be extended to include the NIR/long-wave infrared (LWIR). At resonance, the large field enhancement provided by the SPR [Fig. 3(d)] partially mitigates the non-zero c-GST extinction coefficient, leading to a larger-than-expected transmission efficiency. The SPR origin of the enhancement phenomenon—consistent with EOT—was confirmed by also analyzing the fields in out-of-plane hole cross sections [Figs. 3(e) and 1(f)] and performing simulations with non-plasmonic metal films ( Supplement 1, Fig. S4). In Figs. 3(e) and 1(f), both a-GST (i) and c-GST (ii) states, at resonance, are investigated with experimentally derived refractive index data for both a-GST and c-GST states. The SPR mode couples to the Ag–GST interface inside the hole, leading to an increased transmission response.

**Fig. 4.**
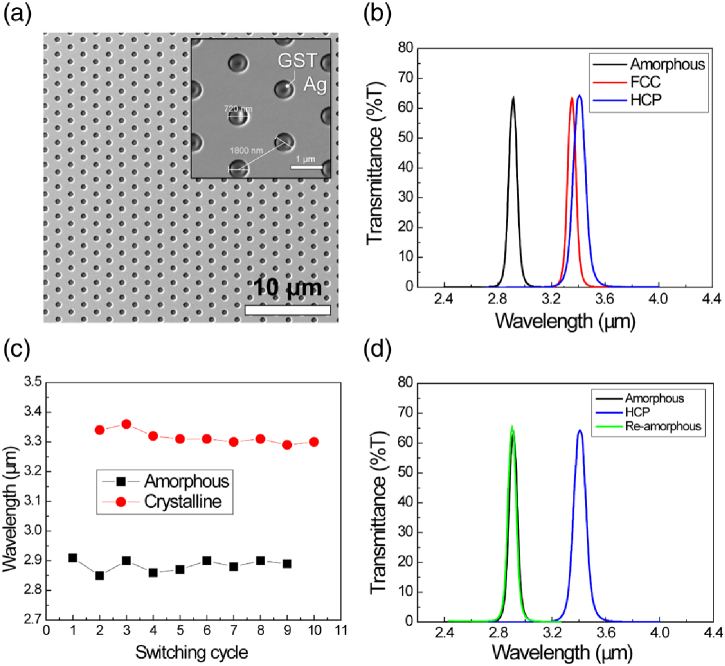
GST-PNA device tuning. (a) SEM micrographs of the fabricated tunable GST-PNA metasurface device showing the full hexagonal array geometry and individual hole morphology (inset). The GST embedded within the Ag PNA can be seen. (b) FTIR (transmission) characterization of the fabricated PNA device showing ∼
70%
 transmission at the resonance and perfect reflection outside the resonance bandwidth. Stability in the spectral response was maintained across many switching cycles; shown through center wavelength reproducibility (c) and spectral shape consistency (d).

The metasurface spectral filter devices were designed for an a-GST resonance at 3.0 µm, which corresponds to a period of 1800 nm (hole diameter 720 nm). A SEM micrograph of one of the GST-PNA devices is shown in Fig. 4(a). 60 nm of Ag was deposited via magnetron sputtering onto $CaF_{2}$, patterned via direct-write photolithography and dry etched to generate the nanohole array; the total patterned device area was 15mm×
15mm (see Supplement 1, Section 3 for further details). 70 nm of GST was then deposited to ensure the nanoholes were completely filled with GST (100% hole volume). This resulted in a <
10nm layer of GST above the Ag surface boundary. FDTD simulations confirm that any ultrathin layer of additional GST has a negligible effect on the device behavior. Last, an encapsulation (capping) layer of $SiO_{2}$ (total thickness <
5nm) was deposited in order to prevent oxidation of the Ag–GST, as well as any partial volatilization that may arise as a result of switching [44]. This layer is fully transparent across the visible-MWIR wavebands. Full details of device characterization comparing the capped/uncapped devices are provided in Supplement 1, Section 2 and Fig. S6.

Transmission-mode Fourier transform IR spectroscopy (FTIR) was used to optically characterize the GST-PNA metasurfaces ( Supplement 1, Section 6). A laser pulse induces crystallization of the as-fabricated a-GST devices; it is then re-characterized in its crystalline state (c-GST). The resulting transmission response due to the respective GST state is shown in Fig. 4(b). FTIR data are shown for both the HCP and FCC phases of c-GST. Samples were subsequently heated by applying a single $160\;mJ\;{cm}^{2}$ 100 ns laser pulse to return to the amorphous phase to be re-characterized. A total of 10 complete phase-change switching cycles was performed, while recording resonance peak position [Fig. 4(c)] to demonstrate the stability of the filtering response. The devices show high stability across all cycles in both the amorphous and crystalline phases, and the shape of the spectral response in each phase is unchanged [Fig. 4(d)], which is a result of the addition of the capping layer. A typical feature of PCMs is thermal hysteresis: the difference between the melting and crystallization temperatures, due to the differing heating and cooling paths. The magnitude of the hysteresis is proportional to the energy loss in the system, which is dependent on PCM stoichiometry, film thickness, laser energy density, and phase-change switching speed. The reversal property in our device is shown to be stable across a number of switching cycles, as shown in Figs. 4(c) and 4(d), with no observable hysteresis, suggesting that irreversible changes in GST-PCM are not significant in our film or device design in contrast to ${VO}_{2}$-based PCM [55].

**Fig. 5.**
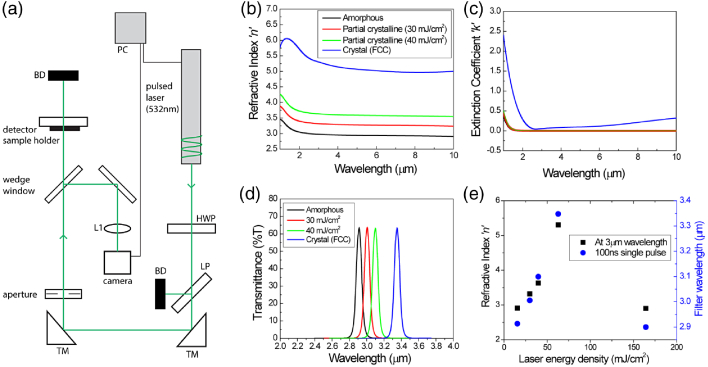
Optically tuned GST-PNA metasurface devices. (a) Setup used for the laser switching demonstration. Complex refractive index measurements (b), (c) of the a-GST, p-GST, and c-GST films (tuned using the all-optical approach), along with the corresponding spectral response of the full GST-PNA metasurface device for each case as experimentally measured via FTIR (d). It can be seen in (e) that with increasing pulse energy, the crystallinity increases until c-GST is achieved. Further increasing the pulse energy allows for the return to a-GST. Upon returning to the amorphous state, the device exhibits nearly identical spectral response to the as-deposited amorphous phase device.

The devices show a transmission efficiency of ∼
70%
 in both crystalline phases, which is among the highest transmission for such a structure reported in the literature, irrespective of spectral band. FWHMs of 74 nm in the amorphous phase and 100 nm in the crystalline phase are in good agreement with simulation results. Moreover, the peak center wavelengths lie at ∼
2.9µ
m in the amorphous state and 3.35 µm in the crystalline state, which are also in good agreement to the simulated values of 3.0 µm and 3.5 µm, respectively. This small deviation is likely the result of either batch-to-batch variation in the refractive index of the deposited GST, microfabrication tolerances, and/or as a result of the addition of the capping layer.

Due to the confined growth circumstances of deposition in nanoholes, the GST inside of the holes likely has a smaller grain size compared to GST deposited on planar substrates. A reduced grain size leads to a larger band gap, and thus less absorption, in the GST inside of the holes. This may explain the low absorption measured in the crystalline phase of the experimentally demonstrated device. This phenomenon suggests that the GST extinction coefficient can be independently optimized based on the GST film growth conditions. Additionally, c-GST grain size is largely influenced by the fluence, wavelength, and pulse duration of the switching-laser, with nanosecond pulses in the ultraviolet regime producing the smallest grain sizes [56]. Therefore, we expect that the lack of c-GST absorption results from a reduced grain size as a result of the confined film growth and the nanosecond optical switching pulse.

### C. Reversible All-Optical Continuous Tuning

For many imaging/spectroscopy applications, active (or rapid) optical tuning is highly desirable, i.e., the passband center wavelength can be reversibly spectrally shifted. This is traditionally achieved through optical, electrical, or thermal stimuli, but an all-solid-state solution has been challenging. Here, we implement an all-optical approach due to the challenges associated with fabricating micro-heater elements across 1-inch diameter optical elements, and subsequently demonstrate continuous spectral tuning of a $∼8.4\;{mm}^{2}$ device with a ns laser pulse. The setup is shown schematically in Fig. 5(a). A 532 nm laser (100 ns) is used to rapidly switch the GST crystallinity, and thus output optical response. The pulse energy, with 7 mm beam diameter, was increased linearly from 2–250 mJ, in order to study the effect of incident pulse energy on the GST crystallinity. A laser spot size of 8.4 mm was chosen due to the 6.5 mm measurement area probed by the FTIR system. By varying the fluence from $10-60\;mJ\;{cm}^{2}$, intermediate (partial crystallinity) phases of GST were generated, with full crystallization being achieved at $∼60\;mJ\;{cm}^{2}$ fluence. Here, three unique partial crystallinity (p-GST) states were formed, with refractive index values ranging from ∼
3.0−
3.75, and Δ
n∼
0.25 between each state. By implementing finer control of the pulse energy (e.g., with a polarizer and half-wave plate) a virtual continuum of p-GST states can be generated (i.e., Δ
n between adjacent states can be minimized, and the number of states increases). In order to return to the amorphous phase, it was found that a fluence of $160\;mJ\;{cm}^{2}$ was required (i.e., reset pulse). Ellipsometry data and FTIR characterization of the optically tuned GST films (a-GST, c-GST, and two p-GST states) are shown in Figs. 5(b)–5(d). The transmission response in each state is approximately constant despite the small increase in extinction coefficient with increasing GST crystallinity, which only slightly increases the FWHM of the fully crystalline state. This is due to the increased field enhancement observed in PNA devices filled with a high-index dielectric medium [38]. As the laser energy density increases, a GST refractive index change is induced through partial phase change, and thus peak resonance spectrally red shifts [Fig. 5(e)]. This behavior is in strong agreement with simulations. Moreover, the a-GST devices—before switching and after laser-induced return (i.e., reset pulse to the amorphous state)—show nearly identical spectral responses, demonstrating stability in the optical tuning mechanism. To the best of our knowledge, this is the first time tunable operation across the MWIR with high transmission efficiency (∼
70%
) and narrowband filtering (∼
70nm FWHM, Q-factor ∼
45) has been achieved.

**Fig. 6.**
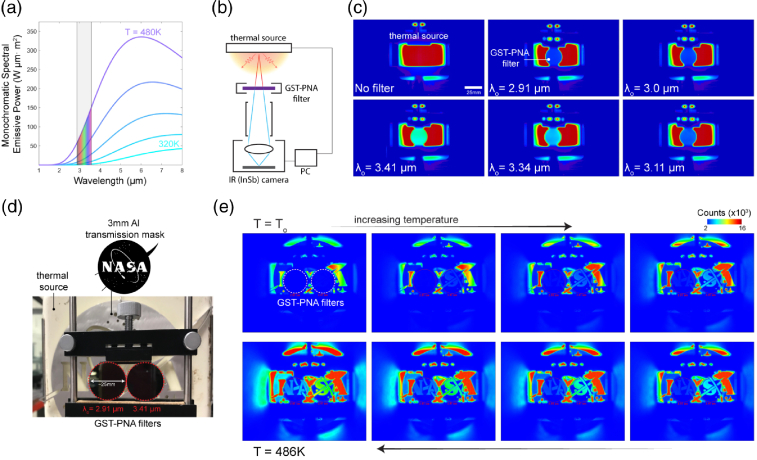
MWIR imaging using tunable GST-PNA metasurface filters. (a) Blackbody thermal emission curves for varying temperature sources with overlaid spectral coverage of the tunable GST-PNA metasurface filters fabricated here. (b) Thermal imaging setup schematic for the results shown in (c), (e); image of setup shown in Supplement 1, Fig. S7. (c) MWIR imaging results at a fixed 486 K hotplate temperature, as a function of varying GST-PNA filter states with varying passband center wavelength, λ
0. (d) RGB image of the setup imaged in (e), which shows the IR image of the same scene, as the temperature of the hotplate is increased from 320 K to 486 K. The left and right filters are centered at 2.91 µm and 3.41 µm, respectively. Variable transmission response through the filters, and subsequent identification of the logo (spatially variant thermal profile), can be observed.

Due to the conformal deposition of the GST films, there is essentially zero heat dissipation in the Ag film as a result of the shallow absorption depth of GST at 532 nm [57]. As a result, the Ag film is not subjected to high temperatures ($T_{m}$: 916.8°C). This, along with the nanosecond timescale of the overall heating/cooling process, prevents the Ag from melting and diffusing into the GST. This lack of Ag diffusion is evidenced by the consistent switching behavior of the device, as Ag atom diffusion into the GST-filled holes would result in absorption and therefore lower transmission, which is not observed in the experimental data. However, for an increasingly large number of phase-change cycles, Ag diffusion mitigation techniques are required because the GST needs to achieve a 635°C melting temperature for phase transition with the mJ-level power laser. To prevent possible diffusion of the Ag into GST after a large number of switching cycles, an atomically thin buffer layer (such as $Al_{2}O_{3}$) can be integrated (through atomic layer deposition or otherwise). This will enable long device lifetimes and stable operation across many switching cycles.

### D. Mid-Wave Infrared Imaging

The thermal radiation from an object is described through its blackbody emission curve, i.e., the cooler an object the longer the peak wavelength of its emission curve. A contact hotplate, for example, with only a relatively small temperature change from 320 K (47°C) to 480 K (213°C) exhibits significantly different spectral emissive power responses [Fig. 6(a)]. To demonstrate real-world imaging applicability, we use our tunable GST-PNA metasurface filters in combination with a contact hotplate (thermal source) and commercial FLIR MWIR camera (imager). A setup schematic is shown in Fig. 6(b) (image of setup shown in Supplement 1, Fig. S7). Fixing the source temperature at ∼
480K, our metasurface filters provide tunable filtering operation across the region of 2.91–3.41 µm [Fig. 6(c)] based on the switching between five different GST-crystalline states (from a-GST to p-GST to c-GST). Because the blackbody emissive power from a 480 K source increases at the longer wavelengths in our targeted spectral region, we expect the tunable filter to provide an increasing transmitted intensity; this is clearly observed in Fig. 6(c). A percentage comparison between expected and experimental transmittance, used to validate our response, is shown in Supplement 1, Fig. S8.

To further demonstrate thermal imaging applicability, in particular to verify transmission homogeneity across the filters through spatial variation of the thermal source, we fix a metal (Al) transmission mask (3 mm thick) with the NASA Insignia logo in front of the thermal source [Fig. 6(d)]. By gradually increasing its temperature (from $T_{0}=320-480\;K$) and optically tuning two separate fabricated GST-PNA filters to exhibit passband center wavelengths of 2.91 µm and 3.41 µm, we are able to show selective MWIR imaging of the logo at two separate passbands simultaneously [Fig. 6(e)]. The accompanying imaging video can be found in Visualization 1.

## 3. DISCUSSION AND CONCLUSION

In summary, we have experimentally demonstrated fully reversible continuously tunable narrowband GST-PNA-based metasurface spectral filters operating across the MWIR by exploiting intermediary partial crystallinities of the phase-change material GST-225. We further show real-time multispectral thermal imaging across the MWIR waveband, by integrating our GST-PNA metasurfaces with a commercial MWIR camera. Our phase-change tunable metasurface filters show best-in-class optical performance, with highest transmission efficiency (∼
70%
) and narrowest bandwidth (∼
74nm), in relation to the wider tunable nanophotonic filter community. This is a direct result of the increased field enhancement, and mitigating the extinction losses of c-GST, arising from the large refractive index contrast of the fully embedded GST within the nanoholes, rather than underneath/above the holes, which results in a low-Q, low transmission, and limited tunability [9,29,35,46,58,59]. Other studies utilizing GST in nanophotonic device architectures have not been experimentally demonstrated in imaging applications, provided sub-optimal filtering performance—specifically a significant widening of the passband upon GST crystallization—and most importantly, lacked experimental demonstration of easily reversible switching capabilities [46]. Moreover, experimental realization of the effect of the inter-hole index has not previously been demonstrated.

Through the utilization of p-GST states—a relatively unexplored concept experimentally—our continuously tunable phase-change metasurfaces represent a fundamentally new spectral filter, opening the door for both high efficiency, high-Q PNA-based metasurface photonic devices. In comparison to other approaches for reconfigurable/tunable filters [3,4,9,11–16,35], the devices here are able to operate continuously across a spectral range of several micrometers (3–5 µm), along with possessing a simple, spectrally agnostic design that is translatable to any waveband. By taking advantage of the fine-tuning provided by optical switching, we have experimentally demonstrated the utility of partially crystalline GST states in order to achieve tuning across the MWIR and shown the tuning is stable across a number of switching cycles. Moreover, after re-analyzing the transmission response of one of the fabricated PNA devices after three months left exposed to the atmosphere (see Supplement 1, Fig. S10 for FTIR data), we have confidence that device longevity—albeit not exhaustively tested—will be within acceptable tolerances for future spectroscopic implementations.

From an applications standpoint, the PNA design is straightforward to manufacture and is capable of switching at ns speeds, making it attractive for systems with time resolution requirements. Through industry standard UV-lithographic and physical vapor deposition techniques, the design scheme for the 1-inch GST-PNA optical elements presented is easily scalable to larger areas. Interestingly, a virtual continuum of crystallinities, thus continuum of passbands, can be achieved by controlling the laser pulse energy. The large pulse energies used here are a result of the relatively long pulse width (100 ns) of the laser used, and the large area ($∼55\;{mm}^{2}$) being switched with a single pulse. Much smaller pulse energies can be used with a shorter pulse duration and/or by employing a raster scanning technique with a reduced beam size to increase the overall fluence [60].

The work presented represents a significant advancement in the state of the art of phase-change tunable metasurface devices/optical components, as well as tunable optical filters. We achieve ∼
70%
 transmission, irrespective of GST phase, and reversible tuning, with devices ∼
1-inch in diameter. This proof of concept demonstration is achieved with a nanosecond pulse laser system; however, the general framework can be translated to a much more compact system for real-world system integration, either optically (see Supplement 1, Section 6 and Fig. S9) or with switching implemented through an electrical stimulus (akin to rewritable optical storage media designs).

Furthermore, the transmission efficiency may be further increased by employing an anti-reflection thin-film coating to each side of the device, or by reducing the extinction coefficient of the GST film. We stress that although GST is opaque in the visible, other phase-change alloys, such as $Sb_{2}S_{3}$, SbTe, GeTe, or a custom blend of chalcogenides such as $Ge_{2}Sb_{2}Se_{4}Te_{1}$, can be used for similar device operation in the visible spectrum [44,61] and thus the general design framework shown here is spectrally agnostic. As a result, we expect the promising findings presented here to be useful not only in fundamental tunable photonic device/metasurface research, but in a host of applications including hyperspectral imaging, remote sensing, and chemical spectroscopy.

## Notes

See Supplement 1 for supporting content.
