# Supplementary material for: Reversible optical tuning of GeSbTe phase-change metasurface spectral filters for mid-wave infrared imaging
Source: Optica. 2020 Jul 1;7(7):746–54. doi: 10.1364/OPTICA.392878 (PMC8262593; doi:10.1364/OPTICA.392878)
Supplement: Supplementary file 1 [file optica-7-7-746-s001.pdf]

Supplemental Document

optica

Reversible optical tuning of GeSbTe phase-change metasurface spectral filters for mid-wave infrared imaging: supplement

Matthew N. Julian,[1,2] **Calum Williams,**[3] **Stephen Borg,**[4] **Scott Bartram,**[4] **and Hyun Jung Kim**[2,4,*]

[1] *Charles L. Brown Department of Electrical and Computer Engineering, University of Virginia, Charlottesville, Virginia 22904, USA*
[2] *National Institute of Aerospace, Hampton, Virginia 23666, USA*
[3] *Department of Physics, Cavendish Laboratory, University of Cambridge, Cambridge, CB3 0HE, UK*
[4] *NASA Langley Research Center, Hampton, Virginia 23666, USA*
[*] *Corresponding author: hyunjung.kim@nasa.gov*

This supplement published with The Optical Society on 1 July 2020 by The Authors under the terms of the Creative Commons Attribution 4.0 License in the format provided by the authors and unedited. Further distribution of this work must maintain attribution to the author(s) and the published article's title, journal citation, and DOI.

Supplement DOI: https://doi.org/10.6084/m9.figshare.12446117

Parent Article DOI: https://doi.org/10.1364/OPTICA.392878

Reversible optical tuning of GeSbTe phase-change metasurface spectral filters for mid-wave infrared imaging

MATTHEW N. JULIAN[1,2], CALUM WILLIAMS[3], STEPHEN BORG[4], SCOTT BARTRAM[4], AND HYUN JUNG KIM[2*]

[1] *Charles L. Brown Department of Electrical and Computer Engineering, University of Virginia, Charlottesville, VA, 22904, USA*
[2] *National Institute of Aerospace, Hampton, VA, 23666, USA*
[3] *Department of Physics, Cavendish Laboratory, University of Cambridge, Cambridge, CB3 0HE, UK*
[4] *NASA Langley Research Center, Hampton, VA, 23666, USA*
[*] *hyunjung.kim@nasa.gov*

This document provides supplementary information to "*Reversible optical tuning of GeSbTe phase-change metasurface spectral filters for mid-wave infrared imaging*" including information about GST deposition and characterization, FDTD simulations, laser characterization and imaging setups, active "read/write" system concepts, and raw FTIR transmission data of the presented filters.

1. Experimental Methods

Thin-film deposition: Double-side polished 25.40 mm diameter (1-inch optics), 1.50 mm-thick calcium fluoride (CaF_2, Esco Optics, Inc) wafers were cleaned in acetone, iso-propanol, and deionized water sequentially, twice, before deposition. The $Ge_2Sb_2Te_5$ (GST) thin films were deposited via RF magnetron sputtering at a base pressure of 2.6 x 10-6 Torr and a deposition pressure of 7 mTorr (10 sccm Ar flow, research-grade, 99.9999% purity). A Ge2Sb2Te5 target (14.3 wt% Ge, 23.8 wt% Sb, 61.9 wt% Te, Mitsubishi materials, Inc.) was sputtered; 50W RF power at room temperature and 10 rpm substrate rotation during deposition. 10 min pre-sputtering was conducted at 7mTorr with 50 sccm of Ar flow with the target shutter open. After 30 minutes of deposition, the growth rates of GST were measured via SEM (VEGA3, TESCAN) as 6.7 nm/min. Prior to XRD and ellipsometry characterization, the as-deposited amorphous phase GST film on CaF2 wafer was annealed at 145°C and 172°C inside the sputtering chamber for 1 hr.

For the XRD and ellipsometry tests, the as-deposited amorphous phase GST film on CaF2 wafer was annealed at 145°C (200°C temperature setting) and 172°C (240°C temperature setting) inside the sputtering chamber for an hour. The substrate temperature of the sputtering system was calibrated by using the thermocouple wafer on the graphite sample holder.

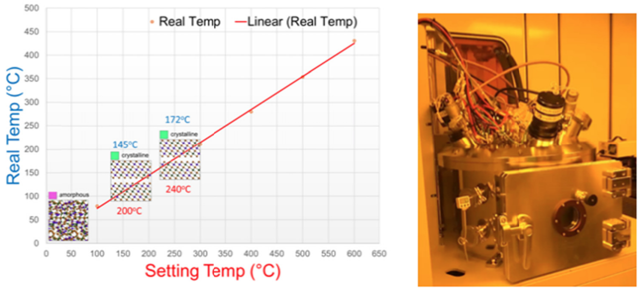

Figure S1: Substrate temperature calibration (left) of the sputtering chamber (photo, right) by using the thermocouple wafer and graphite sample holder.

It is well known that GST will contract slightly when transitioning from amorphous to crystalline phases. These thickness changes were measured so that their effect of PNA device performance could be predicted and understood (effects are shown in the simulation results in Figure S5).

GST composition ratio: The chemical composition of as-deposited GST thin films was determined by DCP-AES (Direct Current Plasma-Atomic Emission Spectroscopy, Luvak, Inc.). The composition was measured as 22 at% Ge, 23.5 at% Sb, and 54.5 at% Te, which is close to a nominal composition of bulk Ge22.2Sb22.2Te55.6. For the DCP-AES test, 1 µm thick GST film was deposited on Si(100) substrate and fully dissolved in 1:1 ratio of hydrochloric acid (HCl) and hydrofluoric acid (HF) to collect and detect the individual Ge, Sb, and Te elements.

Refractive index and extinction coefficient: The complex refractive indices of the bare CaF_2 substrate and the GST-225 film on CaF_2 were measured over the wavelength range of 193 nm -33 µm. The RC2® ellipsometry system (Model: DI, J.A. Woollam) used to measure the properties from 193 nm-1690 nm spectral range with 55° to 75° angles of incidence by 10° step and the Complete EASE v.6.33 used for the data analysis. IR-VASE (Infrared Variable-angle spectroscopic ellipsometry, J.A. Woollam) was used to measure the optical properties from 1.7-33 µm and transmission intensity data from 1.7-13.5µm at angles of incidence of 55° to 75°, increments of 10°. Data analysis was performed using WVASE v3.908 software.

2. FDTD Simulations

Finite Difference Time Domain (FDTD) simulations were performed using a commercial electromagnetic simulator (Lumerical FDTD Solutions). Full 3D simulations for GST-PNA metasurface device geometry were performed using a plane wave (full spectral coverage), symmetric boundary conditions in the x and y dimensions, and perfectly matched layer boundaries in z. A 10 x 10 x 5 nm mesh size was used across the device itself. Simulations and parameter sweeps were allowed to converge for each iteration. Index and E-and-H-field profile monitors were placed at various positions along the z-axis in order to accurately monitor the simulation. A power monitor was placed in the far-field of the device in order to remove any near-field effects that may be present while collecting transmission data. Complex dispersive material models were used for Ag (Johnson and Christy model) and SiO_2 (material data), whereas GST thin-film index models were implemented using experimentally determined values (IR-ellipsometry). Additional FDTD simulations were performed to understand the effect of various parameters of the PNA device performance. Film thickness, metal film material, (hole diameter)/(array period) factor, and reflection spectrum were all simulated in order to optimize the device as well as confirm the role of surface plasmon resonance (SPR) and lack of absorption in the device away from the resonance condition.

	Ge	Sb	Te	

Sample	Chemical composition as at% (atomic ratio)			Melting point (°C)
Lab grown specimen	22 (2)	23.5 (2)	54.5 (5)	611
Stoichio-metric composition	22.2	22.2	55.6	615

Figure S2. Table showing the characteristics of the ideal and lab-grown GST-225 specimen. Samples show near-idealized values for atomic ratio %s as well as melting temperature.

	GST film thickness (nm)			GST film thickness (-%)		
RT	200nm	100nm	25nm			
170□	189nm	94.6nm	24nm	5.5%	5.4%	4%
260□	187nm	94.6nm	22.7nm	6.5%	5.4%	9.2%
360□	185nm	92.7nm	22.7nm	7.5%	7.3%	9.2%

Figure S3. Summary of the change in GST film thickness as measured on a CaF_2 via SEM cross sectional analysis. The film contracts as it becomes more crystalline (i.e. increasing crystalline fractions of p-GST). Contractions of nearly 10% were observed.

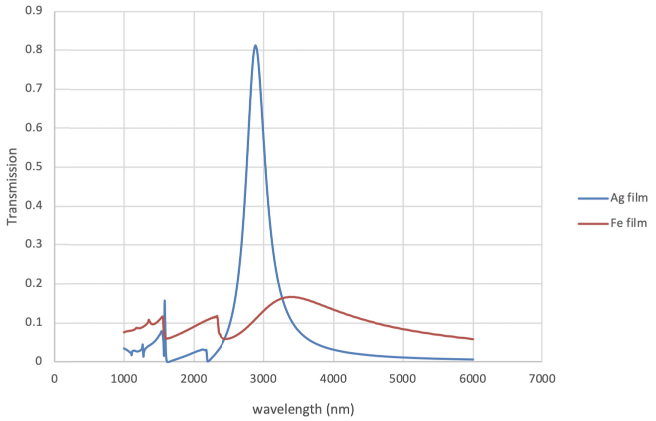

Figure S4. Comparison of an Ag metal film to an Fe metal film (t = 60 nm for both cases) with a-GST filling the holes in the metal films. The Fe film clearly lacks a SPR, as is expected for a non-plasmonic film such as Fe.

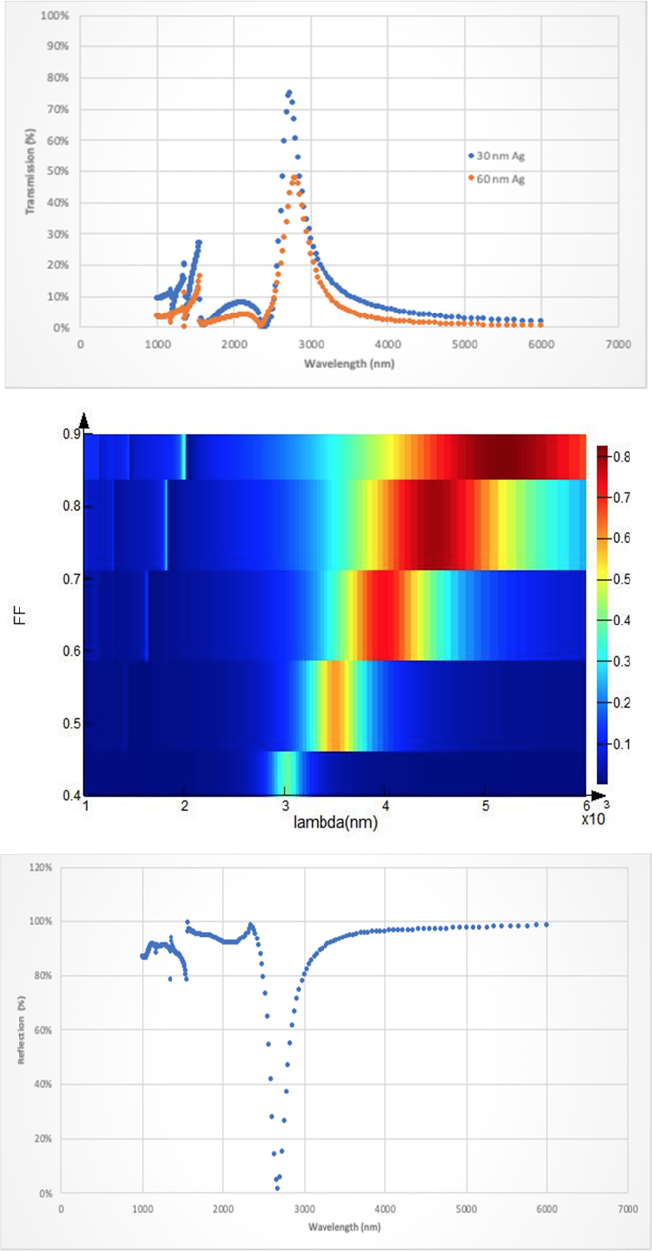

Figure S5. (Top) Effect of GST film thickness on PNA device performance. The contraction of the GST shows no negative effects on the device resonance. Transmission is increased, as expected, due to the reduced absorption length in the thinner GST film. (middle) Fill factor (FF), defined here as the ratio of hole diameter to array period, vs. transmission for the proposed device. As hole diameter increases the transmission also increases (as expected) however, the Q factor is rapidly deteriorated. A ratio of 0.4 was chosen for the device presented in this manuscript. (bottom) Plot of the reflectance spectrum of the presented PNA device showing perfect reflection away from resonance. This confirms a lack of absorption in the resonance.

3. Device Fabrication and Characterization

For the PNA device fabrication, 60 nm Ag film was deposited on CaF_2 substrates by using the RF sputtering at a base pressure of 6.6 x 10^{-6} Torr and a deposition pressure of 3 mTorr. The 14.7 Å/sec slow deposition rate is selected for the precise control of the 60 nm film thickness. ~800 nm thickness AZ1500, high resolution photoresist, was spin coated at 4000 rpm and soft baked at 100°C for 60 s. Direct-write UV-laser lithography (DWL 66fs, Heidelberg Inc.) was used for nanohole patterning with 2-mm writing head. Post-development (AZ 300 MIF developer), Ag thin films were dry-etched using inductively coupled plasma (ICP, TRION Tech.) equipment in CF_4 / Ar mixed-gases while maintaining 15 mTorr pressure, 500 W inductive power, and 150 V DCbias voltage. The GST film was conformally deposited after the nanohole patterned on Ag / CaF_2 sample. AZ100 remover was used as chemical stripper.

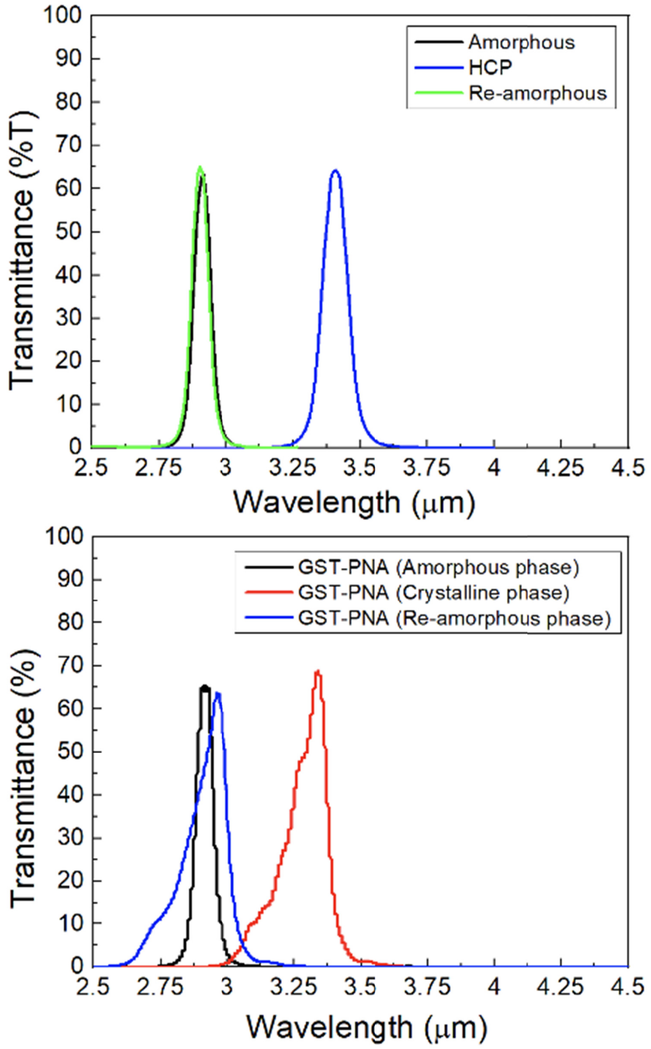

Figure S6. Measured transmission characteristics of the GST-PNA device both with (*top*) and without (*bottom*) the SiO2 capping layer (thickness < 5 nm). Without the capping layer, the resonance shape is dramatically and irreversibly shifted as a result of surface oxidation and volatilization of the GST film. The capping layer mitigates these effects and maintains the resonance quality across switching cycles.

4. Laser Characterization Setup

The laser used is a Quantel Evergreen (PN: EHP2715111) Nd:YAG operating at 532 nm and has 340mJ max energy output. The laser has a flat-top output shape rather than a Gaussian profile. The beam shape characteristics can be measured using a camera. However, to calculate energy density, both energy and beam diameter is needed. The output beam of the laser is 9.82 mm. However, the beam is apertured to 7 mm in order to ensure a uniform spatial profile. The beam is then directed towards the detector (Instrument – PN: FieldMaxII TOP, Accessory – PN: J-50MB-

YAG, Coherent). Using a wedge window 99% of the beam energy is deflected into the energy detector. Energy density can then calculated using the energy from this detector and the diameter of the aperture. The other 1% of the split beam is directed into a CCD detector in order to characterize the beam profile (Newport LBP2-HR-VIS).

5. IR Imaging Demonstration

In order to demonstrate the utility of the presented filters in an imaging system, the setup below was used. The components of the system are described in Figure S7. Although the system in this section is used passively, we describe here a method for implementing real-time tuning of the presented imaging system. In order to evaluate the performance of the filters in the imaging system, a comparison between measured data (in terms of counts at the detector) and expected values based on Planck's Law calculations (Figure S8). The measured data is in strong agreement with expected values.

IR-images were captured using the setup schematically shown in Fig. 5b (photographs of the setup shown in Supplementary Figure S7). An IR camera (FLIR SC8300HD, 0.02 W/m2 minimum pixel sensitivity) was used to record the images. A field stop was used in order to eliminate unwanted signal from polluting the measurement data. A NASA insignia logo was milled into a 15 cm x 15 cm x 3 mm aluminum plate and used in front of a contact hotplate which was used to mimic a blackbody thermal source. The hotplate was heated to 486K and allowed to stabilize for the measurements and images shown in the figure. For the video demonstration (Supplementary Movie 1), the hotplate/object was heated from 320K to 486K.

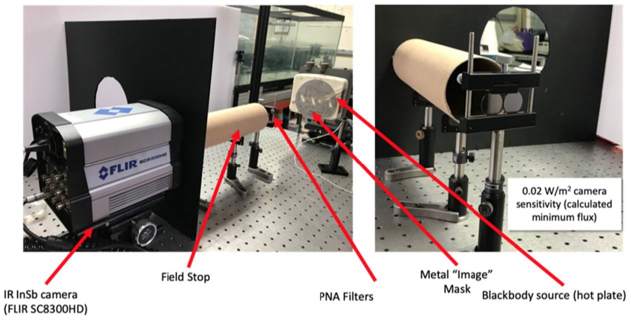

Figure S7: Image of MWIR Imaging Setup
Photograph of the imaging setup used in the manuscript. An InSb MWIR camera is used in conjunction with a field stop to reduce the field of view to include only the desired thermal object (in order to reduce noise in the measurements). Two GST PNA filters are placed in front of the field stop in order to be imaged simultaneously. A hotplate is used as a blackbody radiator, and an Al plate bearing the NASA logo is used as an image mask.

**Blackbody (BB) Temp**	**Center λ_o**	**½ Power - λ_o**	**½ Power +λ_o**	**HPBW**	**BB Emissive Power @ λ_o**	**Filter Transmittance @ λ_o**	**Theory**	**Imager**	**Measured**
°C	µm	µm	µm	µm	W/m²	W/m²	%	Counts	%
213.0	2.910	2.873	2.947	0.074	3.3	2.2		2668.1	
°K	3.000	2.963	3.037	0.074	3.9	2.5	13.8%	3098.2	16.1%
486.15	3.110	3.073	3.147	0.074	4.6	3.0	18.4%	3662.2	18.2%
	3.340	3.303	3.377	0.074	6.2	4.0	34.9%	5034.3	37.4%
T	3.410	3.373	3.447	0.074	6.7	4.4	8.1%	5433.9	7.9%
65%	2.910	2.873	2.947	0.074	3.3	2.2		2668.1	

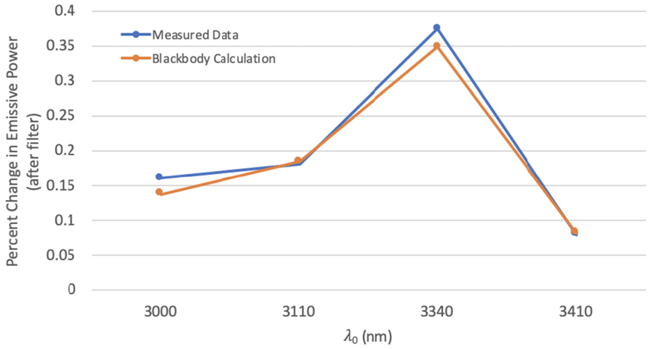

Figure S8: % comparison - BB / exp.
Comparison of the measured spectral irradiance vs. the expected values (after the PNA filter). The plot shows the % change in emissive power for both cases. As can be seen in the plot, the lines are largely overlapping, representing a very low experimental error.

6. Active "Read/Write" System Concept: Laboratory / Real World Systems

Although a laser-based actively tunable imaging system is sufficient for a laboratory environment, for a real-world application a more compact system must be realized. In order to achieve a compact system, a flashlamp can be employed consisting of a number of small, high powered diode sources with short pulse widths (as are commercially available - www.hamamatsu.com/us/en/product/lasers/semiconductor-lasers/plds/index.html). A small flashlamp can also be used, as these are typically quite compact and have large output powers (www.coherent.com/assets/pdf/PulseLife-G-Stack-DataSheet.pdf). Lastly, high-powered, ultra-compact lasers are also commercially available, and could work in a real-world system if integrated similar to a common (albeit higher-power) DVD-RW/CD-RW writer. Figure S9 shows an initial active imaging system, to be implemented in the next steps of this work. Following demonstration, the flashlamp system will be implemented followed by all electrical tuning.

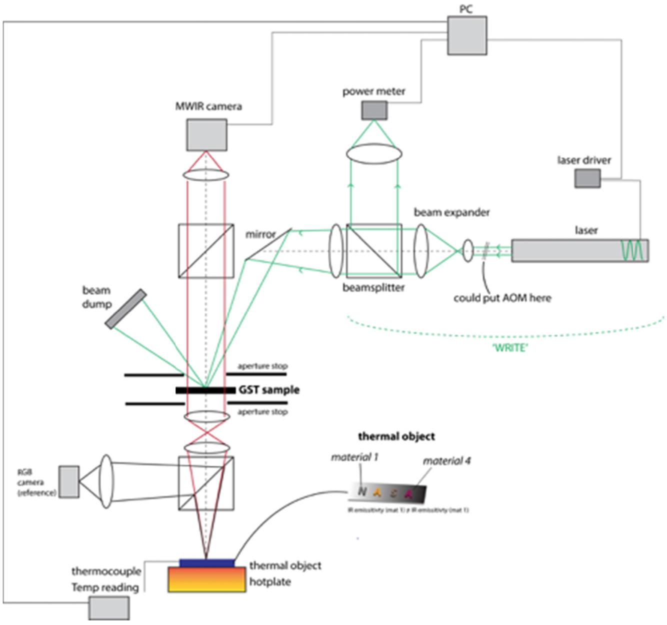

Figure S9: Laboratory-scale Active Imaging System
Schematic of a laboratory-based actively tunable GST PNA thermal imaging system capable of real-time active tuning. The setup is based on the switching setup presented in the main manuscript. In addition to the main "write/rewrite" system, which is now incident on the GST at a non-normal angle, a thermal source is placed behind the GST filter and an IR camera placed in front. In this configuration, simultaneous "read" and "write" functions can be readily realized.

7. FTIR (transmission) characterization of the fabricated PNA devices (amorphous and crystalline phases): re-validation after approx. 3 months

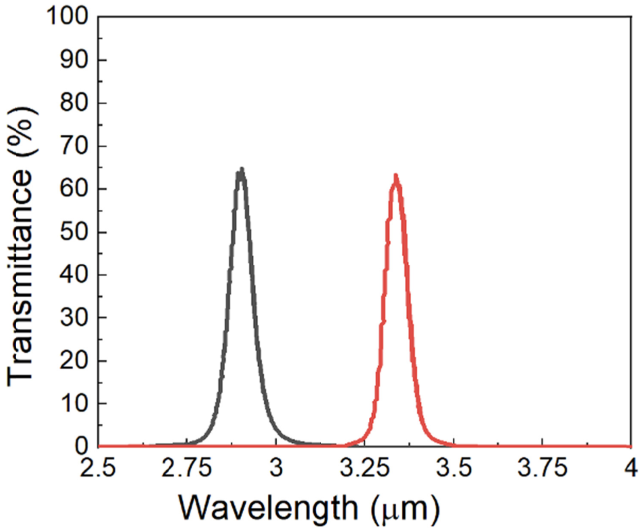

Figure S10: FTIR (transmission) re-characterization of the fabricated PNA devices, amorphous phase with centre wavelength of ~ 2.91 μm (black) and crystalline phase centre wavelength of ~ 3.4 μm (red). Both filters show high transmission efficiency (<70%) and narrowband filtering (~70nm FWHM, Q-factor ~48). Data taken ~3 months after initial fabrication.
